# Deep learning model based on endoscopic images predicting treatment response in locally advanced rectal cancer undergo neoadjuvant chemoradiotherapy: a multicenter study

**DOI:** 10.1007/s00432-024-05876-2

**Published:** 2024-07-13

**Authors:** Junhao Zhang, Ruiqing Liu, Xujian Wang, Shiwei Zhang, Lizhi Shao, Junheng Liu, Jiahui Zhao, Quan Wang, Jie Tian, Yun Lu

**Affiliations:** 1 Department of Gastrointestinal Surgery, The Affiliated Hospital of Qingdao University, No. 16, Jiangsu Road, Shinan District, Qingdao, 266003 China; 2 Graduate School for Elite Engineers, Shandong University, Jinan, China; 3 Department of Gastric and Colorectal Surgery, General Surgery Center, The First Hospital of Jilin University, Changchun, China; 4 CAS Key Laboratory of Molecular Imaging, Institute of Automation, Chinese Academy of Sciences, Beijing, China; 5 Department of Gastroenterology, The Affiliated Hospital of Qingdao University, Qingdao, China; 6 Department of Gastroenterology, Endoscopy Center, The First Hospital of Jilin University, Changchun, China; 7 School of Engineering Medicine, Beihang University, Beijing, 100191 China

**Keywords:** Deep learning, Artificial intelligence, Endoscopy, Neoadjuvant chemoradiotherapy, Rectal cancer, Treatment response

## Abstract

**Purpose:**

Neoadjuvant chemoradiotherapy has been the standard practice for patients with locally advanced rectal cancer. However, the treatment response varies greatly among individuals, how to select the optimal candidates for neoadjuvant chemoradiotherapy is crucial. This study aimed to develop an endoscopic image-based deep learning model for predicting the response to neoadjuvant chemoradiotherapy in locally advanced rectal cancer.

**Methods:**

In this multicenter observational study, pre-treatment endoscopic images of patients from two Chinese medical centers were retrospectively obtained and a deep learning-based tumor regression model was constructed. Treatment response was evaluated based on the tumor regression grade and was defined as good response and non-good response. The prediction performance of the deep learning model was evaluated in the internal and external test sets. The main outcome was the accuracy of the treatment prediction model, measured by the AUC and accuracy.

**Results:**

This deep learning model achieved favorable prediction performance. In the internal test set, the AUC and accuracy were 0.867 (95% CI: 0.847–0.941) and 0.836 (95% CI: 0.818–0.896), respectively. The prediction performance was fully validated in the external test set, and the model had an AUC of 0.758 (95% CI: 0.724–0.834) and an accuracy of 0.807 (95% CI: 0.774–0.843).

**Conclusion:**

The deep learning model based on endoscopic images demonstrated exceptional predictive power for neoadjuvant treatment response, highlighting its potential for guiding personalized therapy.

**Supplementary Information:**

The online version contains supplementary material available at 10.1007/s00432-024-05876-2.

## Introduction

Neoadjuvant chemoradiotherapy followed by surgery has become the standard treatment for locally advanced rectal cancer in clinical practice (Glynne-Jones et al. 2017; Saraf et al. 2022). This treatment approach is capable of inducing tumor regression, achieving complete pathological regression (pCR) in an estimated 20% of patients and improving quality of life and survival outcomes (Koukourakis et al. 2023; Maas et al. 2010). However, the response to treatment varies significantly among individuals. Patients who are less sensitive to neoadjuvant chemoradiotherapy may suffer more from additional toxicity than they benefit, experiencing side effects such as gastrointestinal adverse reactions, sexual dysfunction, urinary system complications, and radiation enteritis (Dossa & Baxter 2023; Koukourakis et al. 2023). Thus, constructing a model to predict treatment response and identify suitable candidates for neoadjuvant treatment has emerged as a hot spot of current research.

In recent years, with the development of deep learning technology, the quantitative features reflecting tumor heterogeneity contained in medical images have been extracted by neural networks and converted into mineable data for decision support analysis (Gadekallu et al. 2022; Gillies et al. 2016). Using neural networks, scholars have attempted to construct radiomic and pathomic models for predicting the response to neoadjuvant chemoradiotherapy treatment in patients with locally advanced rectal cancer (Bulens et al. 2020; Wan et al. 2022). Although the utility of MRI images and digital pathological slice images has been proven by numerous studies, it must be acknowledged that the utilization of MRI images often necessitates the manual delineation of regions of interest (ROI), and pathological images require a complex preprocessing protocol before analysis (Feng et al. 2022; Zhang et al. 2020). Consequently, a new image type is needed to solve the above limitations.

Endoscopic images are becoming increasingly valued for their ability to directly visualize tumor morphology and capture a broad spectrum of details highlighting the heterogeneity of tumors, including key characteristics such as size, shape, and texture, all of which are of significant interest for image analysis (Ignjatovic et al. 2009). Moreover, it has overcome the inherent limitations of MRI and pathological images, as it offers easy access and eliminates the need for complex preprocessing, saving a significant amount of time and cost. In the management of locally advanced rectal cancer, endoscopic images have been applied to evaluate tumor regression after neoadjuvant therapy, thereby providing guidance for implementing a watch and wait approach (Thompson et al. 2023; Wang et al. 2023). However, the potential of these images to predict tumor regression prior to the initiation of neoadjuvant therapy and thus aid in the selection of suitable candidates for this treatment remains underexplored.

This study aimed to develop a deep learning model based on pre-treatment endoscopic images to predict tumor regression in locally advanced rectal cancer patients who underwent neoadjuvant chemoradiotherapy.

## Methods

### Ethical approval

This study was conducted in accordance with the *Declaration of Helsinki* and approved by the ethics committees of The Affiliated Hospital of Qingdao University (no. QYFY WZLL 27,925) and The First Hospital of Jilin University (no. 2023-KS-201). Informed consent was waived due to the retrospective nature of the study.

### Study design and participants

**Fig. 1:**
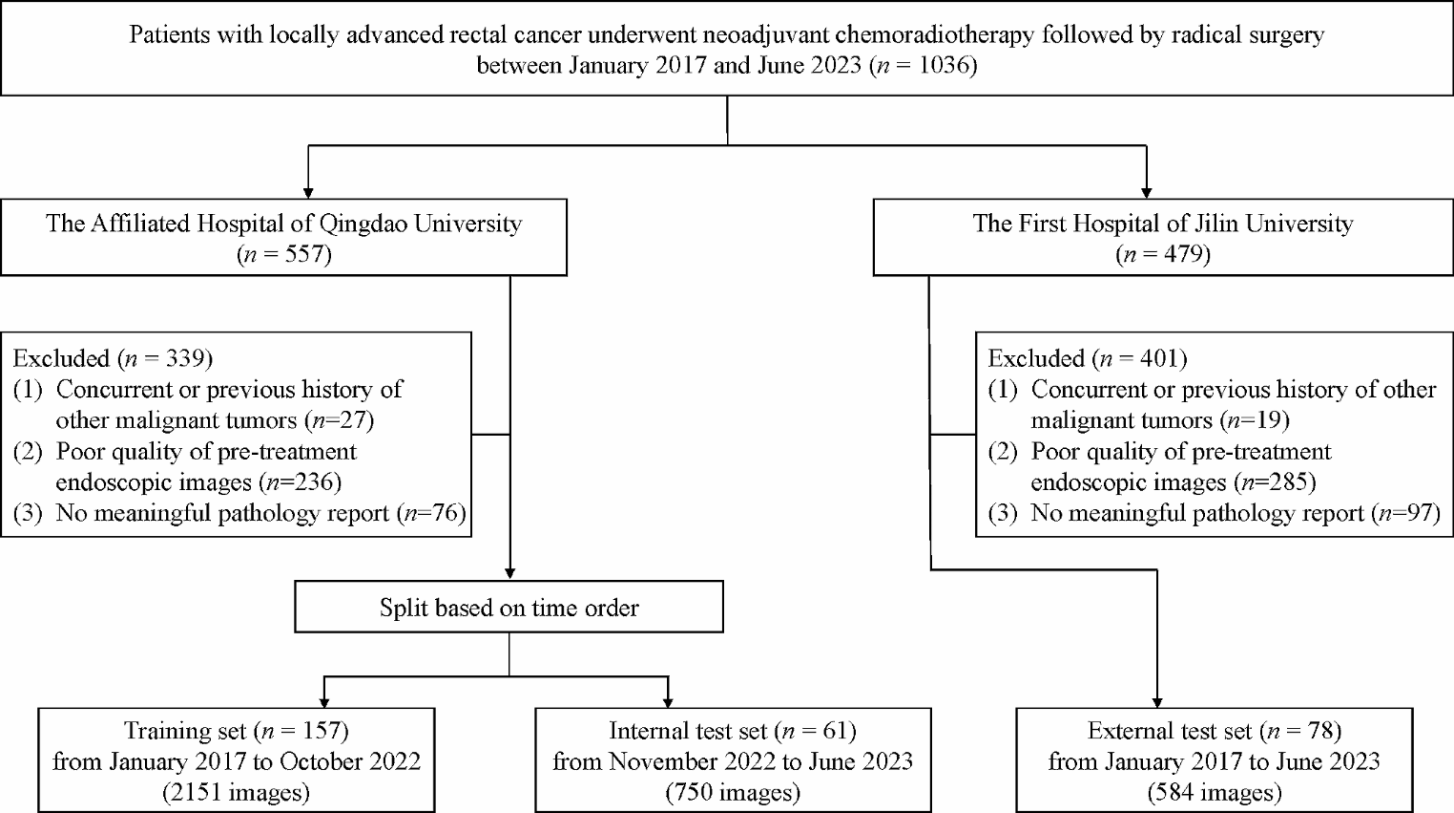
Flowchart of patient enrollment for training and validation of the endoscopic image-based deep learning model

In this study, we retrospectively recruited patients with locally advanced rectal cancer who visited two prominent Chinese medical centers, The Affiliated Hospital of Qingdao University and The First Hospital of Jilin University, from January 2017 to June 2023. All patients received neoadjuvant chemoradiotherapy after a multidisciplinary consultation, and we obtained endoscopic images from colonoscopy examinations conducted within 1–2 weeks before the start of their neoadjuvant treatment. The data from The Affiliated Hospital of Qingdao University were allocated to a training set (January 2017 to October 2022) and an internal test set (November 2022 to June 2023) based on the time order, with a ratio of 5:2, while the data from The First Hospital of Jilin University served as an independent external test set for validating the performance of the prediction model. The inclusion and exclusion criteria were identical for both medical centers to ensure consistency. The inclusion criteria were as follows: (1) locally advanced rectal cancer patients with adenocarcinoma confirmed by histopathology; (2) received standard neoadjuvant chemoradiotherapy; (3) underwent radical surgery after neoadjuvant chemoradiotherapy; and (4) pre-treatment endoscopic images available. The exclusion criteria were as follows: (1) concurrent or previous history of other malignant tumors; (2) poor quality of pre-treatment endoscopic images; and (3) lacked meaningful pathological information (Fig. 1).

### Neoadjuvant chemoradiotherapy regimens

All the eligible participants received standard long-course concurrent chemoradiotherapy before surgery: (1) long-course radiotherapy (45–54 Gy in 25–30 fractions to the pelvis) with capecitabine 825 mg/m^2^ po bid, Monday–Friday, on each day that radiotherapy was given throughout the duration of radiotherapy (typically 28–30 treatment days depending on stage); (2) long-course radiotherapy (45–54 Gy in 25–30 fractions to the pelvis) with 5-FU 225 mg/m^2^ IV over 24 h daily on days 1–5 or days 1–7 for 5 weeks; or (3) long-course radiotherapy (45–54 Gy in 25–30 fractions to the pelvis) with 5-FU 400 mg/m^2^ IV bolus + leucovorin 20 mg/m^2^ IV bolus for 4 days during week 1 and 5 of radiotherapy.

### Assessment of treatment response

Treatment response was evaluated by the tumor regression grade (TRG) from the postoperative pathology report, (Chen et al. 2021) and the pathology report was meticulously examined by a seasoned pathologist with substantial clinical expertise. The TRG was evaluated based on the 8th American Joint Committee on Cancer (AJCC) cancer staging manual: TRG 0 was defined as no remaining viable cancer cells; TRG 1 was defined as only small clusters or single cancer cells remaining; TRG 2 was defined as residual cancer remaining but with predominant fibrosis; and TRG 3 was defined as minimal or no tumor kill with extensive residual cancer (Chen et al. 2021). To effectively stratify patients, this study employed a binary outcome variable, combining TRG 1 patients who exhibited a positive response to neoadjuvant chemoradiotherapy without achieving complete tumor regression with TRG 0 patients for analysis. TRG 0 and TRG 1 were categorized as good response (GR), whereas TRG 2 and TRG 3 were categorized as non-good response (non-GR) (Zhang et al. 2020).

### Data acquisition and preprocessing

The clinical baseline data of the participants were obtained from the doctor workstations of each medical center, and white light endoscopic images were obtained from the endoscopy center of each medical center. Endoscopic images from the training and internal test sets were captured using one of the following Olympus endoscopic instruments: CF-H290l, GIF-Q260J, or PCF-Q260Jl. All of these devices were manufactured by Olympus Corporation in Tokyo, Japan. For the independent external test set, endoscopic data were collected using one of the following devices: Olympus, PCF-H290l, Tokyo, Japan; SonoScape, EC-550, Shenzhen, China; or PENTAX, EC-3840 M, Tokyo, Japan. The meaningful endoscopic images were selected by two gastroenterologists with more than ten years of clinical experience using Adobe Photoshop 2022 (Adobe, San Jose, CA, USA).

### Model development

**Fig. 2:**
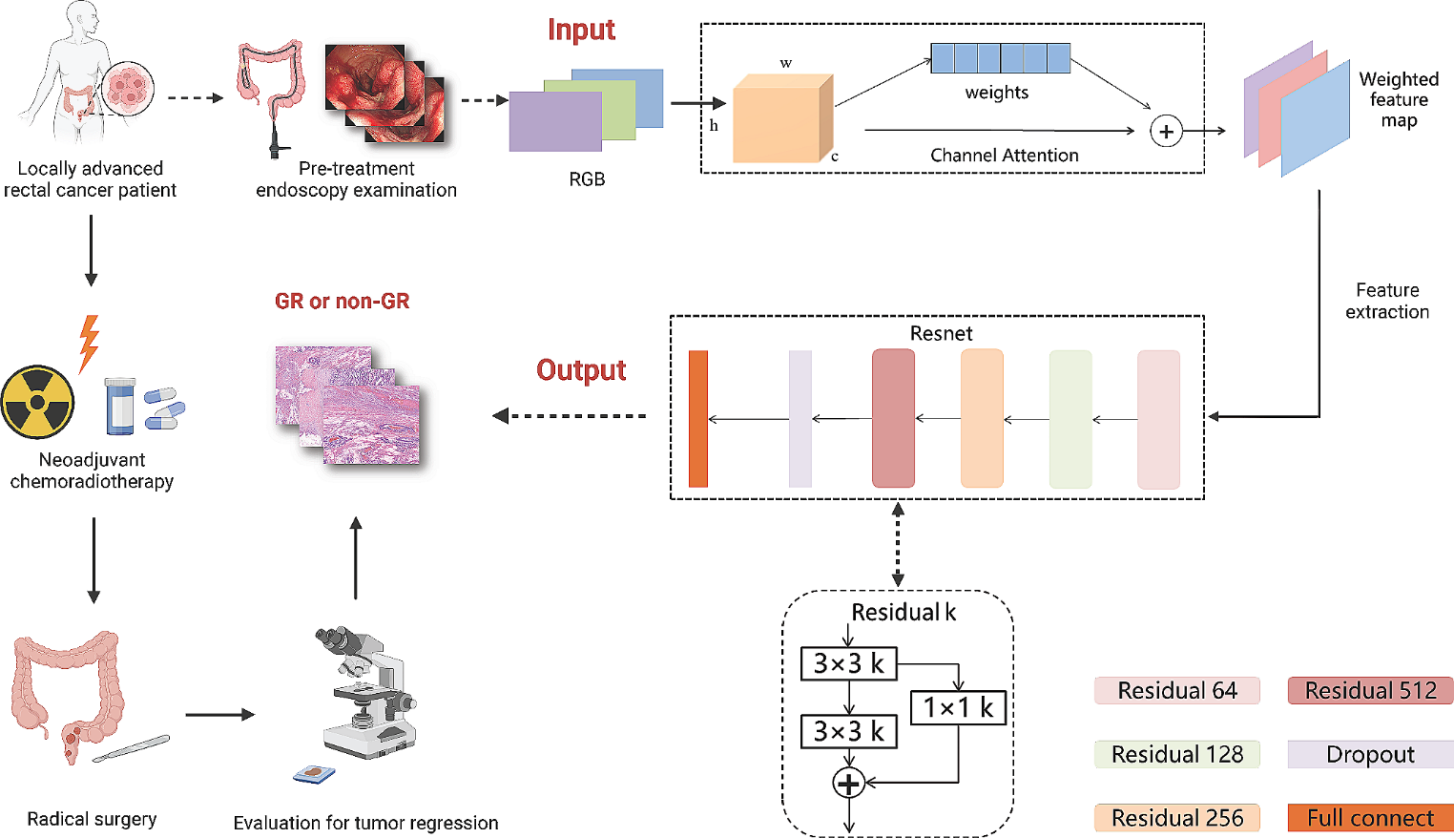
Workflow and network architecture of the endoscopic image-based deep learning model. The Residual k refers to the number of channels in each layer, which can vary from 64 to 128, 256, or 512. GR: Good response

First, the designed model was trained and evaluated on the internal dataset and then tested on the independent external test set. Specifically, the internal dataset was divided into training and internal test sets based on time order at a ratio of 5:2. The training set was further split into a training subset (80%) and a validation subset (20%) for training and optimizing the model. Subsequently, the channel attention-ResNet model was proposed. Channel attention was employed before ResNet to prioritize the channel dimension of the image, namely, the relationship between different color channels, and to assist the model in focusing on features in the image that were related to the predicted categories. The overall architecture of the proposed model is illustrated in Fig. 2. The input consisted of RGB three-channel endoscopic images, and the output was the prediction of treatment response status.

During the analysis and processing of endoscopic images, due to variations in image sizes, the input endoscopic images were first resized (each image was resized to 1920 × 1080). Then, channel attention was employed to assign different weights to different channels, enabling the network to focus on more critical areas during subsequent prediction. Next, the ResNet structure was used to predict and discriminate images.

Specifically, a convolutional module was initially utilized to extract high-dimensional image features. Four residual modules were subsequently added to extract more sophisticated image features while preserving low-dimensional feature information. To address overfitting caused by a limited dataset, a dropout layer was introduced after the residual blocks. The extracted features were ultimately passed through a fully connected layer to generate the final prediction probability for GR. Following the acquisition of prediction probabilities, for clearer interpretation, we performed binary classification on the output results of this prediction model using a threshold of 0.5.

Furthermore, adaptive moment estimation was used for optimizing the model, with a batch size of 10 and a dropout rate of 0.5. To mitigate the overfitting issue, an early stopping strategy was incorporated, in which the model was made to stop training when the loss of validation was at its minimum. To ensure the accuracy of the model, a cross-entropy loss function was incorporated to calculate the model’s prediction loss, guiding the model optimization toward correct predictions. The cross-entropy loss function was defined as follows:

$$\eqalign{& L(\theta ) = \cr & - {1 \over m}\sum\limits_{i = 1}^m {{\rm{[}}{y^{{\rm{(i)}}}} \cdot {\rm{log(}}{{\mathop y\limits^ \wedge }^{{\rm{(i)}}}}{\rm{) + (1 - }}{y^{{\rm{(i)}}}}{\rm{)}} \cdot {\rm{log(1 - }}{{\mathop y\limits^ \wedge }^{{\rm{(i)}}}}{\rm{)]}}} \cr}$$

where *m* represents the number of samples,*y*^(*i*)^ represents the true label of the *i*th sample, and ${\hat y^{(i)}}$ represents the predicted label of the *i*th sample.

### Heatmap generation

To visualize the model’s analysis of image features, the Gradient-weighted Class Activation Mapping (Grad-CAM) algorithm was used to construct image heatmaps for visualizing the importance of different regions in GR prediction. Specifically, we saved the feature map of the last convolutional layer of the proposed model and obtained the weight scores of different regions through the class confidence scores generated by the model; these scores were multiplied and subsequently added to obtain the class significance map.

### Statistical analysis

The clinical baseline characteristics of the participants were compared using the *t* test for normally distributed continuous variables, the Mann‒Whitney *U* test for skewed continuous variables and the χ^2^ test for categorical variables. The prediction performance of the deep learning model was assessed by the area under the curve (AUC), accuracy, sensitivity, specificity, positive predictive value (PPV) and negative predictive value (NPV). Medians and 95% confidence intervals (CIs) of these performance measures were calculated by bootstrapping. Furthermore, a calibration curve was generated to assess the agreement between the deep learning model predictions and the actual observations at different percentiles of the predicted probabilities. Univariate and multivariate logistic regression analyses were conducted to investigate the factors associated with GR. Only the variables with statistical significance at the level of 0.05 in univariate analyses were included in multivariate analyses for clinical prediction model development. All the statistical analyses were two-sided, and *p* < 0.05 was considered indicative of statistical significance. All the statistical analyses were performed using SPSS (version 25.0; IBM Corporation, Armonk, NY, USA) and R (version 4.3.0). All the experiments were carried out on an Ubuntu system with an NVIDIA GeForce 1080Ti GPU and a CUDA 10.2 with lr = 1e-5. Specifically, Python (version 3.7) was used with PyTorch (version 3.7), the scikit-learn package (version 0.21.3) and the matplotlib package (version 3.3.2).

## Results

### Baseline characteristics

This multicenter retrospective observational study included 296 patients with locally advanced rectal cancer who underwent neoadjuvant chemoradiotherapy followed by radical surgery. Of these, 218 participants from The Affiliated Hospital of Qingdao University were assigned to a training set (*n* = 157, age = 60.88 ± 10.33 years) and an internal test set (*n* = 61, age = 62.08 ± 10.04 years). The remaining 78 participants from The First Hospital of Jilin University (*n* = 78, age = 54.17 ± 10.62 years) served as the external test set. The GR rates of the training set, internal test set and external test set were 28.7%, 34.4% and 29.5%, respectively. The distributions of GRs and non-GRs were not significantly different among the training set, the internal test set and the external test set (*p* = 0.70). Detailed information regarding the TRG ratio in each dataset is provided in the Supplemental Materials. The clinical characteristics of these patients are shown in Table 1.

**Table 1:** Patient characteristics in the training, internal test and external test sets

Variables	Training set (*n* = 157)			Internal test set (*n* = 61)			External test set (*n* = 78)		
	GR (*n* = 45)	non-GR (*n* = 112)	*p*-value	GR (*n* = 21)	non-GR (*n* = 40)	*p*-value	GR (*n* = 23)	non-GR (*n* = 55)	*p*-value
Age(years)	62.29 ± 8.72	60.31 ± 10.89	0.28	63.38 ± 9.69	61.40 ± 10.27	0.47	50.26 ± 13.57	55.80 ± 8.74	0.08
Sex			0.68			0.15			0.165
Male	34 (75.6%)	81 (72.3%)		12 (57.1%)	30 (75.0%)		15 (65.2%)	44 (80.0%)	
Female	11 (24.4%)	31 (27.7%)		9 (42.9%)	10 (25.0%)		8 (34.8%)	11 (20.0%)	
BMI (kg/m^2^)	23.70 ± 2.78	24.45 ± 3.25	0.18	23.17 ± 3.90	23.72 ± 3.16	0.52	22.37 ± 3.48	23.11 ± 1.98	0.348
Pretreatment CEA level (ng/mL)	2.66 (1.55–6.10)	3.95 (2.01–8.62)	0.027	1.86 (1.39–3.02)	3.43 (2.14–5.75)	0.002	5.12 (2.79–32.59)	5.23 (2.09–20.89)	0.483
Pretreatment clinical T stage			0.21			0.09			0.30
cT1	3 (6.7%)	1 (0.9%)		0 (0%)	0 (0%)		0 (0%)	0 (0%)	
cT2	1 (2.2%)	2 (1.8%)		1 (4.8%)	0 (0%)		1 (4.3%)	0 (0%)	
cT3	35 (77.8%)	90 (80.4%)		12 (57.1%)	32 (80.0%)		11 (47.8%)	27 (49.1%)	
cT4	6 (13.3%)	19 (17.0%)		8 (38.1%)	8 (20.0%)		11 (47.8%)	28 (50.9%)	
Pretreatment clinical N stage			0.552			0.47			0.115
cN0	2 (4.4%)	2 (1.8%)		0 (0%)	2 (5.0%)		3 (13.0%)	1 (1.8%)	
cN1	9 (20.0%)	19 (17.0%)		6 (28.6%)	8 (20.0%)		4 (17.4%)	13 (23.6%)	
cN2	34 (75.6%)	91 (81.3%)		15 (71.4%)	30 (75.0%)		16 (69.6%)	41 (74.5%)	
Tumor differentiation			0.01			0.14			< 0.001
Well	5 (11.1%)	1 (0.9%)		2 (9.5%)	0 (0%)		5 (21.7%)	0 (0%)	
Moderate	36 (80.0%)	101 (90.2%)		17 (81.0%)	35 (87.5%)		16 (69.6%)	38 (69.1%)	
Poor	4 (8.9%)	10 (8.9%)		2 (9.5%)	5 (12.5%)		2 (8.7%)	17 (30.9%)	
Postoperative T stage			< 0.001			< 0.001			< 0.001
ypT0	10 (22.2%)	0 (0%)		15 (71.4%)	0 (0%)		11 (47.8%)	0 (0%)	
ypT1	7 (15.6%)	3 (2.7%)		1 (4.8%)	2 (5.0%)		3 (13.0%)	1 (1.8%)	
ypT2	17 (37.8%)	27 (24.1%)		3 (14.3%)	11 (27.5%)		4 (17.4%)	11(20.0%)	
ypT3	9 (20.0%)	74 (66.1%)		2 (9.5%)	26 (65.0%)		5 (21.7%)	38 (69.1%)	
ypT4	2 (4.4%)	8 (7.1%)		0 (0%)	1 (2.5%)		0 (0%)	5 (9.1%)	
Postoperative N stage			0.029			0.087			0.004
ypN0	36 (80.0%)	65 (58.0%)		19 (90.5%)	26 (65.0%)		21 (91.3%)	30 (54.5%)	
ypN1	8 (17.8%)	37 (33.0%)		2 (9.5%)	11 (27.5%)		0 (0%)	18 (32.7%)	
ypN2	1 (2.2%)	10 (8.9%)		0 (0%)	3 (7.5%)		2 (8.7%)	7 (12.7%)	
Interval to surgery* (days)	68.73 ± 23.34	65.53 ± 32.23	0.490	77.90 ± 32.99	76.30 ± 38.64	0.872	69.61 ± 26.18	78.73 ± 37.09	0.288
Tumor size^†^ (mm)	51.62 ± 19.79	56.59 ± 16.37	0.108	55.61 ± 17.32	52.92 ± 19.99	0.604	59.39 ± 21.33	61.80 ± 22.96	0.668
Distal margin from anal verge^‡^ (mm)	55.01 ± 19.97	63.80 ± 30.23	0.035	53.61 ± 23.54	66.24 ± 28.46	0.087	50.35 ± 20.07	56.35 ± 22.35	0.27

Data were shown as mean ± standard deviation for normal distributed continuous variables, median (25th percentile and 75th percentile) for skew continuous variables, or number (%) for categorical variables. ^*^The time interval from the completion of the last neoadjuvant chemoradiotherapy to the time of radical surgery. ^†^Tumor size was measured by the distance between the upper and lower margins of the tumor, assessed using MRI. ^‡^The distal margin from the anal verge was determined through MRI measurements. BMI: Body mass index; GR: Good response

**Fig. 3:**
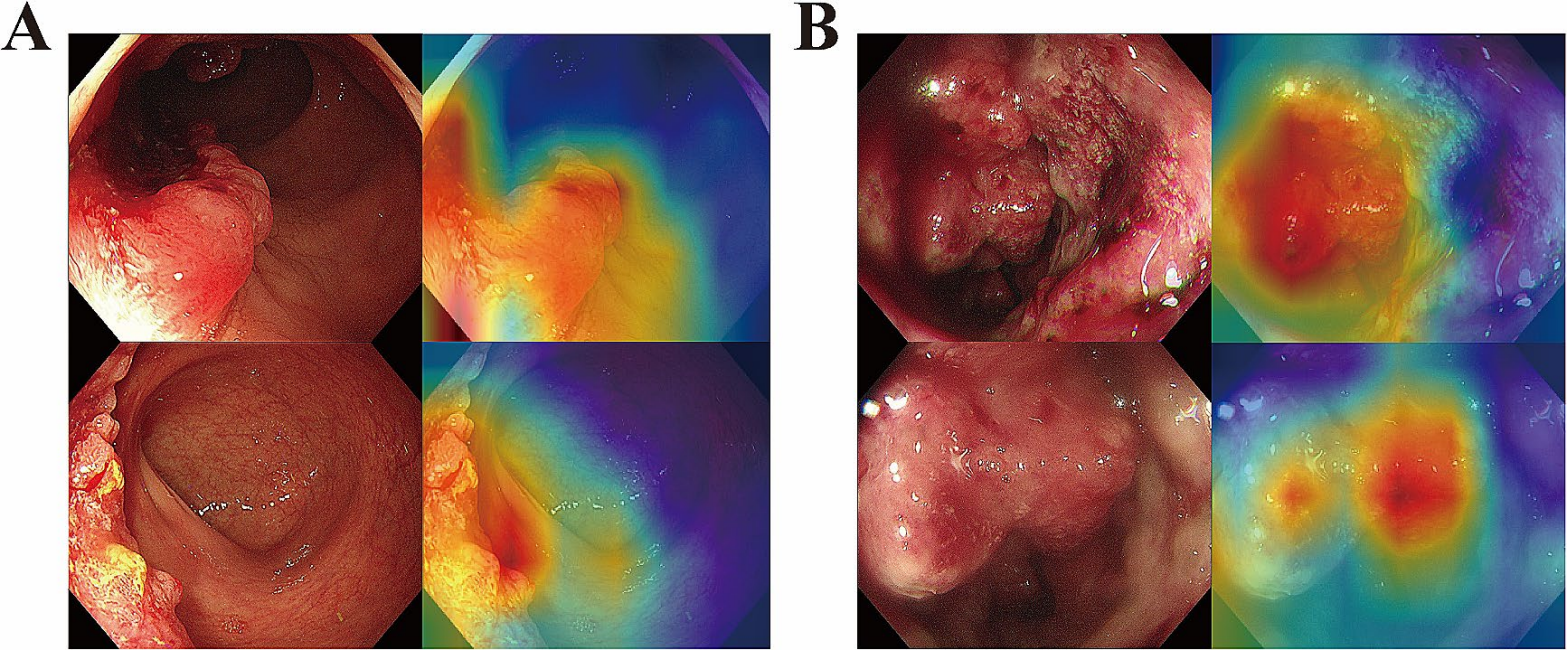
t-SNE analysis of endoscopic images from the GR and non-GR groups. GR: Good response

This study included a total of 3485 endoscopic images from 296 patients. Specifically, the training set comprised of 2151 images from 157 patients, the internal test set included 750 images from 61 patients, and the external test set consisted of 584 images from 78 patients. Of these 3485 endoscopic images, 1007 were from the GR group and 2478 were from the non-GR group. To conduct a qualitative evaluation of endoscopic images from these two groups, t-distributed stochastic neighbor embedding (t-SNE) was employed for visualization analysis. Specifically, features from the last fully connected layer of an untrained model were reduced to two dimensions using t-SNE with data from the internal test set. The results indicate that distinguishing between the features of the original images from the GR and non-GR groups is challenging (Fig. 3).

### Visual interpretation of the heatmap

**Fig. 4:**
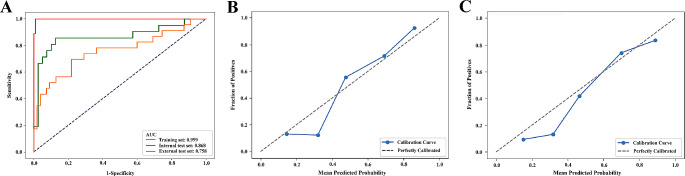
Endoscopic images and corresponding heatmaps of two patients with tumor regression. (**A**) 54-year-old female patient, GR; (**B**) 68-year-old male patient, non-GR. Endoscopic images from different views provided complementary information for treatment response prediction. GR: Good response

Based on Grad-CAM, visual heatmaps were created to elucidate the image recognition mechanism of a deep learning model for endoscopic images. Figure 4 shows the recognized endoscopic images of two patients, GR and non-GR patients, along with their corresponding heatmaps. The weights in the heatmap increased progressively from blue to green to yellow to red (Selvaraju et al. 2017). A deeper red color in the heatmap signified a higher weight, indicating that the specific region of the original image contributed more significantly to the neural network’s ability to predict the treatment response. The most valuable location on endoscopic images was the inner region of the tumor.

### Ablation results of the channel attention

An ablation analysis was conducted to evaluate the efficiency of the channel attention module. The results indicated that incorporating channel attention improved the model’s performance, with the AUC of the internal test set increasing by 11.4% and the AUC of the external test set increasing by 4.7%. The detailed information is shown in the Supplemental Material.

### Performance of the endoscopic image-based deep learning model

**Fig. 5:**
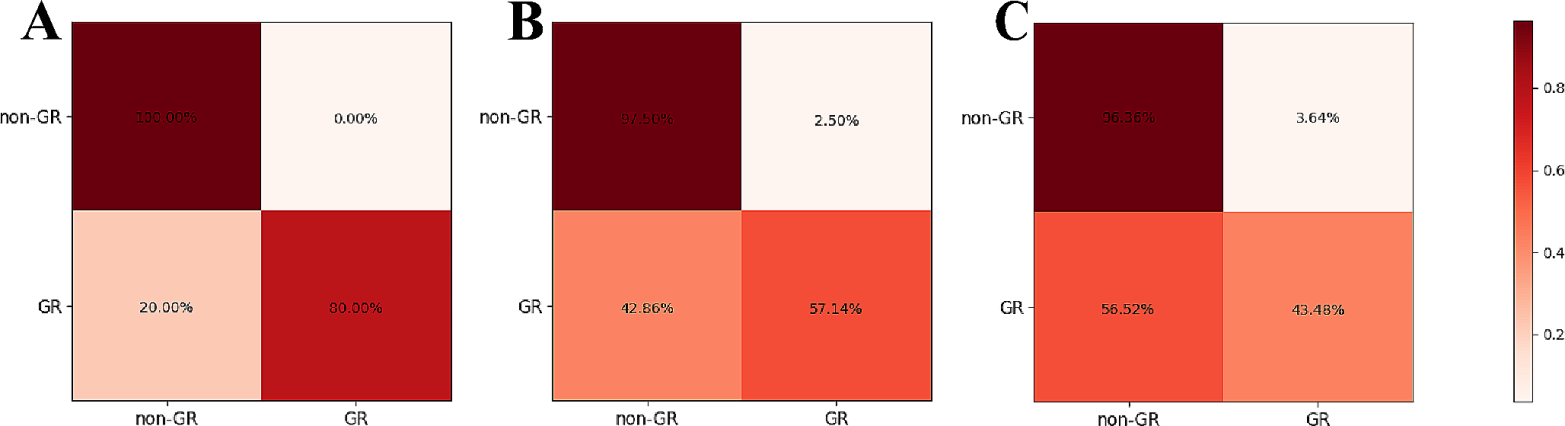
Receiver operating characteristic (ROC) curves and calibration curves of the prediction model based on endoscopic images. (**A**) ROC curves of the training, internal test, and external test sets. (**B**) Calibration curve of the internal test set; (**C**) Calibration curve of the external test set. The calibration curves show the agreement between the predicted and observed treatment response outcomes

**Fig. 6:**
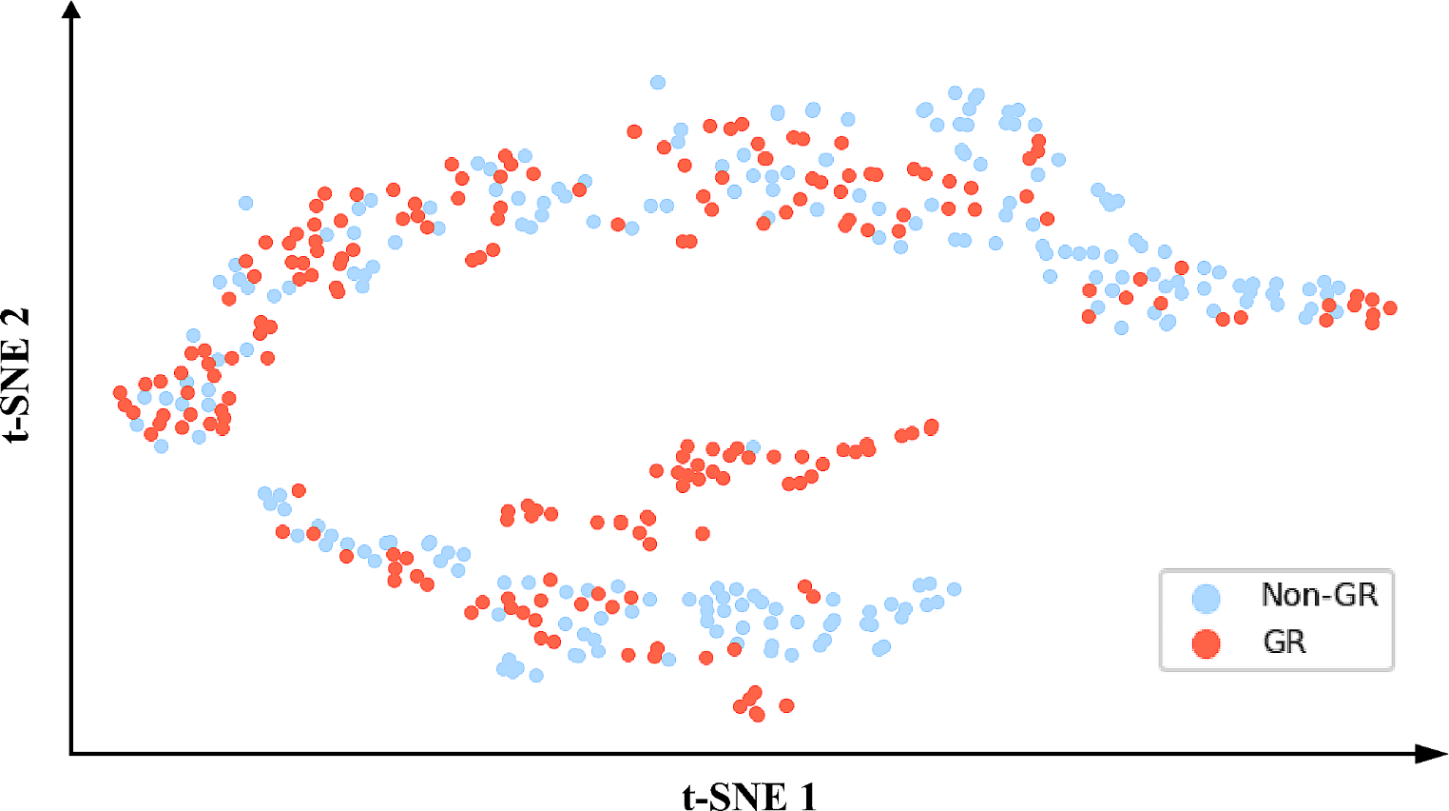
Normalized confusion matrix of the endoscopic image-based deep learning model. (**A**) Training set; (**B**) Internal test set; (**C**) External test set. True and predicted subtype classifications are shown on the y- and x-axes, respectively, such that the correct predictions are shown on the diagonal from the top left to the bottom right of each matrix. The red gradient represents the model accuracy for detecting each subtype. The darker the red color is, the better the model performance. GR: Good response

The endoscopic image-based prediction model demonstrated excellent predictive ability in the internal test set (AUC: 0.867, 95% CI: 0.848–0.941) and the external test set (AUC: 0.758, 95% CI: 0.724–0.834). The receiver operating characteristic (ROC) curves are shown in Fig. 5A. The accuracy reached 0.836 [95% CI: 0.818–0.896] in the internal test set and 0.807 [95% CI: 0.774–0.843] in the external test set. The specificity of the deep learning model was remarkably high in both test sets (0.963–0.975), while the sensitivity was approximately 0.500. The PPV and NPV in both test sets exceeded 0.800, with the PPV in the internal test set being even greater at 0.923 [95% CI: 0.862–0.971] (Table 2). The normalized confusion matrix of the endoscopic image-based deep learning models is shown in Fig. 6. The calibration curves of the endoscopic image-based deep learning model for treatment response prediction showed good agreement between the prediction and actual treatment response status in the internal and external test sets (Fig. 5B and C). Although the calibration curves of both the internal and external test sets did not perfectly align with the ideal curve, the results of the Hosmer–Lemeshow test for both the internal test set (*χ*^*2*^ = 0.143, *p* = 0.980) and the external test set (*χ*^*2*^ = 0.143, *p* = 0.980) indicated that the differences observed were not statistically significant.

**Table 2:** Performance of the endoscopic image-based deep learning model

	AUC	Accuracy	Sensitivity	Specificity	PPV	NPV
Training set (*n* = 157)	0.999(0.996,1.000)	0.942(0.906,0.974)	0.800(0.671,0.909)	1.000(0.999,1.000)	1.000(0.999,1.000)	0.925(0.886,0.965)
Internal test set (*n* = 61)	0.868(0.848,0.941)	0.836(0.818,0.896)	0.571(0.513,0.747)	0.975(0.952,0.988)	0.923(0.862,0.971)	0.812(0.801,0.886)
External test set (*n* = 78)	0.758(0.724,0.834)	0.807(0.774,0.843)	0.435(0.337,0.563)	0.963(0.934,0.970)	0.833(0.708,0.853)	0.803(0.783,0.846)

Data were shown as mean (95% confidence interval). AUC: Area under the curve; NPV: Negative predictive value; PPV: Positive predictive value

In addition to the model discrimination and calibration discussed earlier, it is essential to consider indicators such as the F1-score and Kappa value when evaluating the performance of deep learning models that deal with imbalanced samples. In the internal test set, the F1-score reached 0.706, and the Kappa value reached 0.601. However, in the external test set, these two metrics were slightly lower than those in the internal test set, with an F1-score of 0.571 and a Kappa value of 0.463 (Table 3).

**Table 3:** F1-score and Kappa value of the endoscopic image-based deep learning model

	Training set (*n* = 157)	Internal test set (*n* = 61)	External test set (*n* = 78)
F1-Score	0.889	0.706	0.571
Kappa	0.851	0.601	0.463

Furthermore, a clinical prediction model based on training set data was developed for comparison with an endoscopic image-based deep learning model. The clinical prediction model demonstrated significantly inferior performance compared to the endoscopic image-based model, with an AUC of 0.555 in the training set. Further information regarding the univariate and multivariate regression analyses, as well as the ROC curve of the clinical prediction model, is shown in the Supplemental Materials.

## Discussion

In this multicenter study, we developed and validated an endoscopic image-based deep learning model for predicting tumor regression in patients with locally advanced rectal cancer who underwent neoadjuvant chemoradiotherapy followed by radical surgery. This model showed encouraging predictive performance and holds potential for personalized neoadjuvant therapy in patients with locally advanced rectal cancer. For patients predicted to have a higher probability of GR, we can recommend standard neoadjuvant treatment to induce tumor regression, aiming for complete pathological regression and achieving organ preservation (Dossa et al. 2017). For patients predicted to have a lower probability of GR, alternative treatment options, such as proceeding directly with radical surgery, followed by adjuvant chemotherapy, or neoadjuvant immunotherapies and molecular targeted therapies based on genetic testing results, can be selected.

Neoadjuvant chemoradiotherapy has become the first-line treatment for locally advanced rectal cancer, (Ludmir et al. 2017) as more studies have shown that it can significantly improve the disease-free survival and overall survival rates of patients (Hall & Smith 2023). However, it is important to acknowledge that not all patients are suitable candidates for neoadjuvant chemoradiotherapy, as some may not benefit because of potential side effects. A comprehensive study conducted by Downing Amy et al. revealed that rectal cancer patients who underwent preoperative neoadjuvant radiotherapy experienced poorer health-related quality of life and higher rates of postoperative complications compared to those who did not receive radiotherapy. These complications included poor bowel control (43.6% vs. 33.0%, odds ratio [OR] = 1.55), severe urinary incontinence (7.2% vs. 3.5%, OR = 1.69), and severe sexual difficulties (34.4% vs. 18.3%, OR = 1.73) (Downing et al. 2019). Therefore, identifying the factors that influence the efficacy of neoadjuvant therapy and selecting suitable patients for this treatment are crucial.

In recent years, medical imaging research has expanded across various fields, including radiology, pathology, and ultrasonography. This research has transformed raw imaging data into valuable insights for disease progression, outcome, and related factor investigation (Huang et al. 2023; Jiang et al. 2023; Skrede et al. 2020; Zhou et al. 2023). Gastrointestinal endoscopy, a widely used medical imaging technique, has emerged as an important source of disease information due to its ability to capture microscopic morphological details that reflect tumor heterogeneity. Notably, advancements in convolutional neural networks have substantially improved the computer-aided diagnosis of gastrointestinal polyps and the classification of benign and malignant growth (Ahmad et al. 2019; Du et al. 2022; Okagawa et al. 2022). Based on these findings, scholars in several studies have attempted to develop a tumor regression prediction model based on endoscopic images after neoadjuvant chemoradiotherapy in patients with locally advanced rectal cancer. This model helps identify patients who achieve complete pathological regression, supporting the use of the “watch and wait” strategy (Garcia-Aguilar et al. 2022). Lan et al. developed a deep learning model to predict tumor regression based on post-treatment endoscopic images that showed an AUC of 0.77 and an accuracy of 0.87 in an independent test set, indicating some clinical importance (Chen et al. 2022). Thompson et al. also created a VGG-19 deep learning model based on endoscopic images from multiple stages during neoadjuvant treatment that achieved an AUC of 0.83 in the test set. Despite the small sample size, the VGG-19 model provided some guidance for dynamically evaluating tumor regression (Thompson et al. 2023). The studies mentioned above rely on post-treatment endoscopic images, which provide direct information on tumor regression or residual after treatment. Therefore, these studies cannot estimate the treatment response earlier or guide personalized treatment accordingly. Therefore, we proposed a model that utilized pre-treatment endoscopic images, enabling the prediction of treatment response at baseline (within 2 weeks following tumor diagnosis) and promoting the early formulation of personalized treatment regimens.

In our study, the channel attention mechanism and ResNet were used to construct a prediction model based on endoscopic images. Unlike the conventional convolutional neural network, ResNet with channel attention can be used to adjust image features, preserve relevant features by assigning appropriate weights, remove irrelevant features, and focus subsequent network blocks on important areas. This approach greatly enhanced the performance of the model, which was validated robustly across internal and external test sets and achieved an AUC of 0.758 and an accuracy of 0.807 in the external test set, these results were slightly lower than those obtained for the internal test set but still satisfactory.

Ultimately, a user-friendly subsystem developed based on this model will be embedded into endoscopy systems for predicting treatment response. The prediction subsystem embedded in the endoscopy system is allowed to directly access endoscopic image data. Model inference is performed on either CPU or GPU platforms to generate treatment response predictions, that is, the probability of GR. The predicted results are subsequently displayed in the user interface for clinicians to reference during the decision-making process.

Additionally, our study had several limitations. First, this study focused solely on the short-term outcome of tumor regression following neoadjuvant chemoradiotherapy in locally advanced rectal cancer patients while neglecting crucial long-term outcomes such as overall survival and disease-free survival, which reflect patients’ long-term prognoses and should be considered in future studies. Second, due to its retrospective design and relatively small sample size, the study may have involved selection bias. Although the model’s predictive performance was validated by an independent external test set, future prospective studies with larger sample sizes are needed to further improve the quality of this study. Third, in this study, only single-modal models based on either endoscopic images or clinical data were developed. We will consider developing a multimodal model that integrates endoscopic images, MRI images, pathological biopsy whole-slide images, and clinicopathological data to optimize patient data utilization and improve prediction performance (Boehm et al. 2022).

In conclusion, the proposed endoscopic image-based deep learning model achieved high accuracy in predicting treatment response in locally advanced rectal cancer patients who underwent neoadjuvant chemoradiotherapy and showed the potential for tailoring neoadjuvant treatment for patients with locally advanced rectal cancer.

## Electronic supplementary material

Below is the link to the electronic supplementary material.

Supplementary Material 1

## Data Availability

The data that support the findings of this study were from The Affiliated Hospital of Qingdao University and The First Hospital of Jilin University. The original data were not publicly available and could only be shared with permission from the ethics committees of The Affiliated Hospital of Qingdao University and The First Hospital of Jilin University.

## Abbreviations

AJCC: American Joint Committee on Cancer
AUC: Area under the curve
CI: Confidence interval
GR: Good response
Grad-CAM: Gradient-weighted Class Activation Mapping
NPV: Negative predictive value
OR: Odds ratio
PPV: Positive predictive value
pCR: Complete pathological regression
ROC: Receiver operating characteristic
ROI: Regions of interest
TRG: Tumor regression grade
