# Supplementary material for: Deep learning model based on endoscopic images predicting treatment response in locally advanced rectal cancer undergo neoadjuvant chemoradiotherapy: a multicenter study
Source: J Cancer Res Clin Oncol. 2024 Jul 13;150(7):350. doi: 10.1007/s00432-024-05876-2 (PMC11246300; doi:10.1007/s00432-024-05876-2)

**Supplemental Material**

**Journal:** Journal of Cancer Research and Clinical Oncology

**Title:** Deep learning model based on endoscopic images predicting treatment response in locally advanced rectal cancer undergo neoadjuvant chemoradiotherapy: A multicenter study

**Correspondence:** Yun Lu, Department of Gastrointestinal Surgery, The Affiliated Hospital of Qingdao University, E-Mail: luyun@qdu.edu.cn

**Supplemental Table 1** TRG ratio of locally advanced rectal cancer patients in the training, internal test and external test sets.

|  | **Training set**  **(*n*=157)** | **Internal test set**  **(*n*=61)** | **External test set**  **(*n*=78)** |
| --- | --- | --- | --- |
| TRG 0 | 10 (6.4%) | 15 (24.6%) | 13(16.7%) |
| TRG 1 | 35 (22.3%) | 6 (9.8%) | 10 (12.8%) |
| TRG 2 | 88 (56.0%) | 35 (57.4%) | 48 (61.5%) |
| TRG 3 | 24 (15.3%) | 5 (8.2%) | 7 (9.0%) |

Data were shown as *n* (%). TRG: Tumor regression grade.

**Supplemental Table 2** Results of channel attention ablation analysis

|  |  | **AUC** | **Accuracy** | **Sensitivity** | **Specificity** | **PPV** | **NPV** | **F1-Score** | **Kappa** |
| --- | --- | --- | --- | --- | --- | --- | --- | --- | --- |
| ResNet | Internal test set | 0.779 | 0.771 | 0.429 | 0.950 | 0.818 | 0.760 | 0.563 | 0.427 |
|  | External test set | 0.724 | 0.769 | 0.348 | 0.946 | 0.727 | 0.776 | 0.471 | 0.346 |
| Channel attention  -ResNet | Internal test set | 0.868 | 0.836 | 0.571 | 0.975 | 0.923 | 0.812 | 0.706 | 0.601 |
|  | External test set | 0.758 | 0.807 | 0.435 | 0.963 | 0.833 | 0.803 | 0.571 | 0.463 |

**Supplemental Table 3** Factors associated with good response in locally advanced rectal cancer underwent neoadjuvant chemoradiotherapy

| **Variables** | **Univariate regression** | | | **Multivariate regression** | | |
| --- | --- | --- | --- | --- | --- | --- |
|  | **z** | **OR (95%CI)** | ***P*–value** | **z** | **OR (95%CI)** | ***P*–value** |
| Age (years) | 1.08 | 1.02 (0.98–1.06) | 0.279 | **-** | **-** | **-** |
| BMI (kg/m^2^) |  |  |  |  |  |  |
| 18.5–24 |  | Reference |  |  |  |  |
| <18.5 or >24 | –0.94 | 0.72 (0.36–1.44) | 0.348 | **-** | **-** | **-** |
| Pretreatment CEA level (ng/mL) |  |  |  |  |  |  |
| 0–5 |  | Reference |  |  |  |  |
| >5 | –1.68 | 0.52 (0.24–1.12) | 0.094 | **-** | **-** | **-** |
| Sex |  |  |  |  |  |  |
| male |  | Reference |  |  |  |  |
| female | –0.41 | 0.85 (0.38–1.87) | 0.679 | **-** | **-** | **-** |
| cT |  |  |  |  |  |  |
| 4 |  | Reference |  |  |  |  |
| 3 | 0.41 | 1.23 (0.45–3.34) | 0.682 | **-** | **-** | **-** |
| 2 | 0.35 | 1.58 (0.12 – 20.69) | 0.726 | **-** | **-** | **-** |
| 1 | 1.81 | 9.50 (0.83–109.23) | 0.071 | **-** | **-** | **-** |
| cN |  |  |  |  |  |  |
| 2 |  | Reference |  |  |  |  |
| 1 | 0.53 | 1.27 (0.52–3.07) | 0.599 | **-** | **-** | **-** |
| 0 | 0.97 | 2.68 (0.36–19.76) | 0.334 | **-** | **-** | **-** |
| Tumor differentiation |  |  |  |  |  |  |
| poor |  | Reference |  |  | Reference |  |
| moderate | –0.19 | 0.89 (0.26–3.02) | 0.853 | –0.19 | 0.89 (0.26–3.02) | 0.853 |
| well | 2.03 | 12.50  (1.09–143.43) | 0.042 | 2.03 | 12.50  (1.09–143.43) | 0.042 |
| Distal margin from  anal verge^*^ (mm) | –1.77 | 0.99 (0.97–1.00) | 0.077 | **-** | **-** | **-** |
| Tumor size^†^ (mm) | –1.60 | 0.98 (0.96–1.00) | 0.109 | **-** | **-** | **-** |

^*^ The distal margin from the anal verge was determined through MRI measurements. †Tumor size was measured by the distance between the upper and lower margins of the tumor, assessed using MRI.

**Supplemental Figure 1** ROC curve of the clinical prediction model in training set


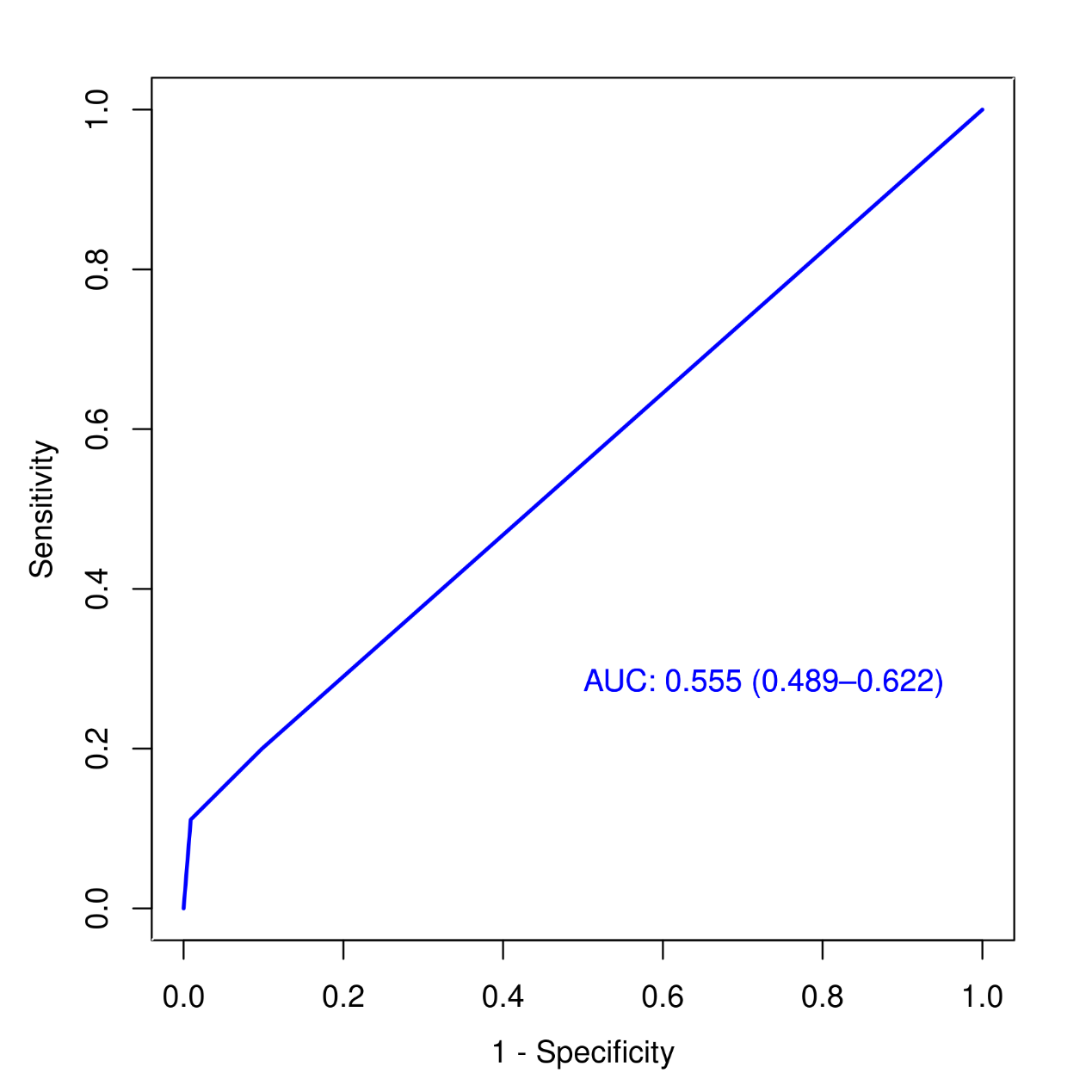

Supplement: Supplementary file 1 — Supplementary Material 1 [file 432_2024_5876_MOESM1_ESM.docx]
